# Supplementary figures and images for: Hexokinase regulates Mondo-mediated longevity via the PPP and organellar dynamics
Source: eLife. 2025 Aug 11;12:RP89225. doi: 10.7554/eLife.89225 (PMC12339002; doi:10.7554/eLife.89225)

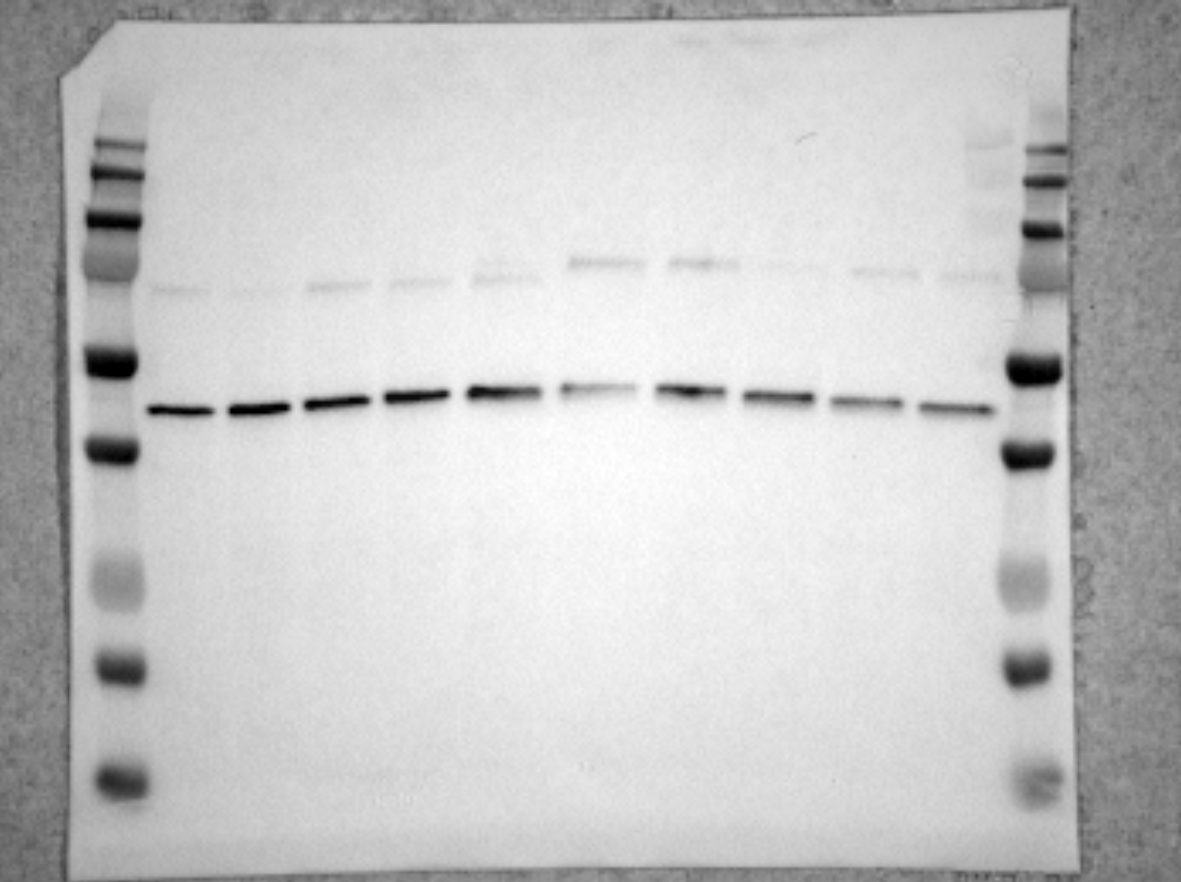

Supplement: Figure 1—figure supplement 1—source data 1. [file elife-89225-fig1-figsupp1-data1.zip › Figure 1-figure supplement 1D-source data 1 - Original files for western blot analysis displayed in Figure 1-figure supplement 1D/Figure 1-figure supplement 1D - Anti-actin for HXK-1-mKate2 and HXK-2-mKate2.tif]

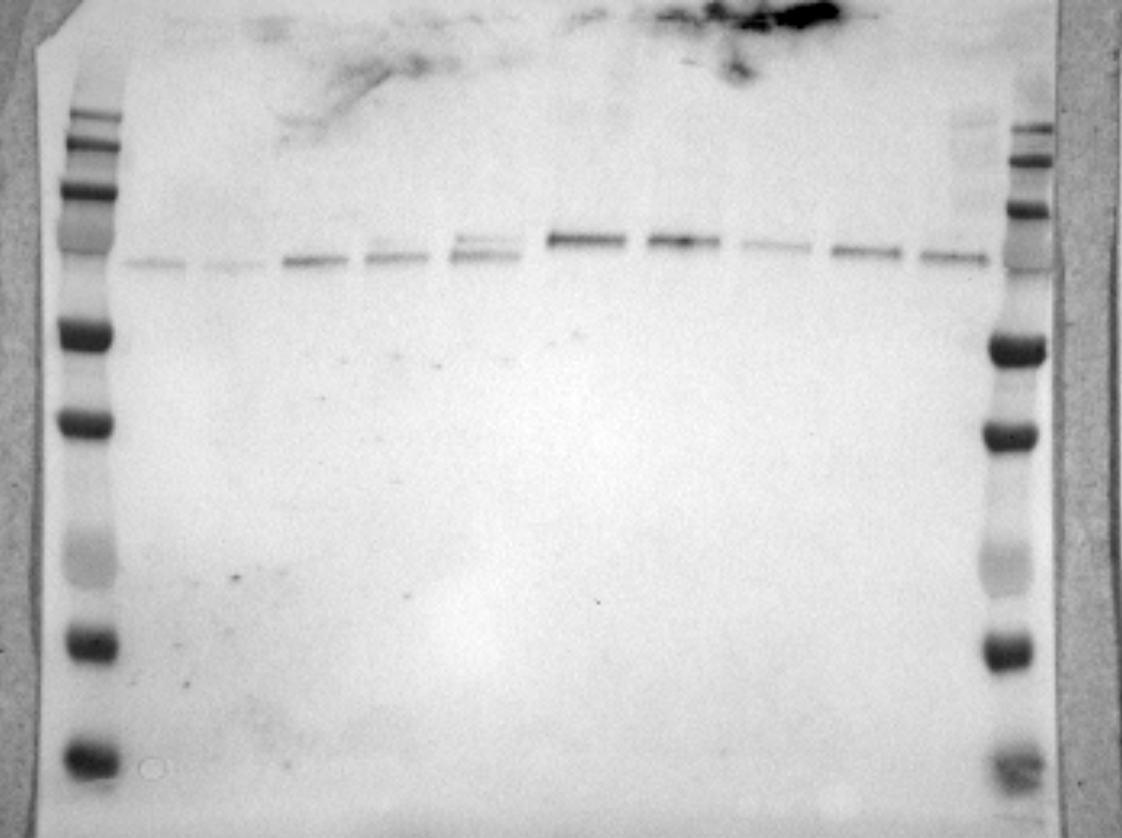

Supplement: Figure 1—figure supplement 1—source data 1. [file elife-89225-fig1-figsupp1-data1.zip › Figure 1-figure supplement 1D-source data 1 - Original files for western blot analysis displayed in Figure 1-figure supplement 1D/Figure 1-figure supplement 1D - Anti-RFP for HXK-1-mKate2 and HXK-2-mKate2.tif]

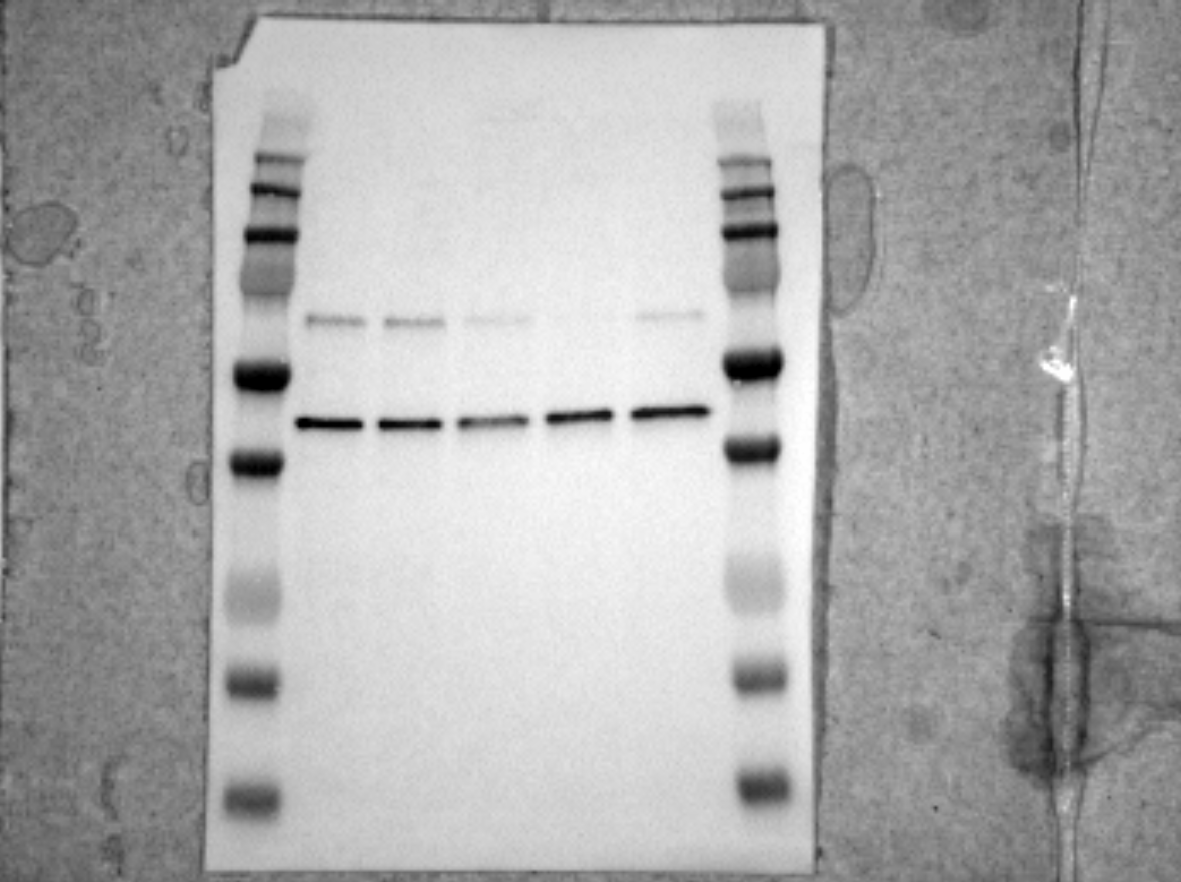

Supplement: Figure 1—figure supplement 1—source data 1. [file elife-89225-fig1-figsupp1-data1.zip › Figure 1-figure supplement 1D-source data 1 - Original files for western blot analysis displayed in Figure 1-figure supplement 1D/Figure 1-figure supplement 1D - Anti-Actin for HXK-3-mKate2.tif]

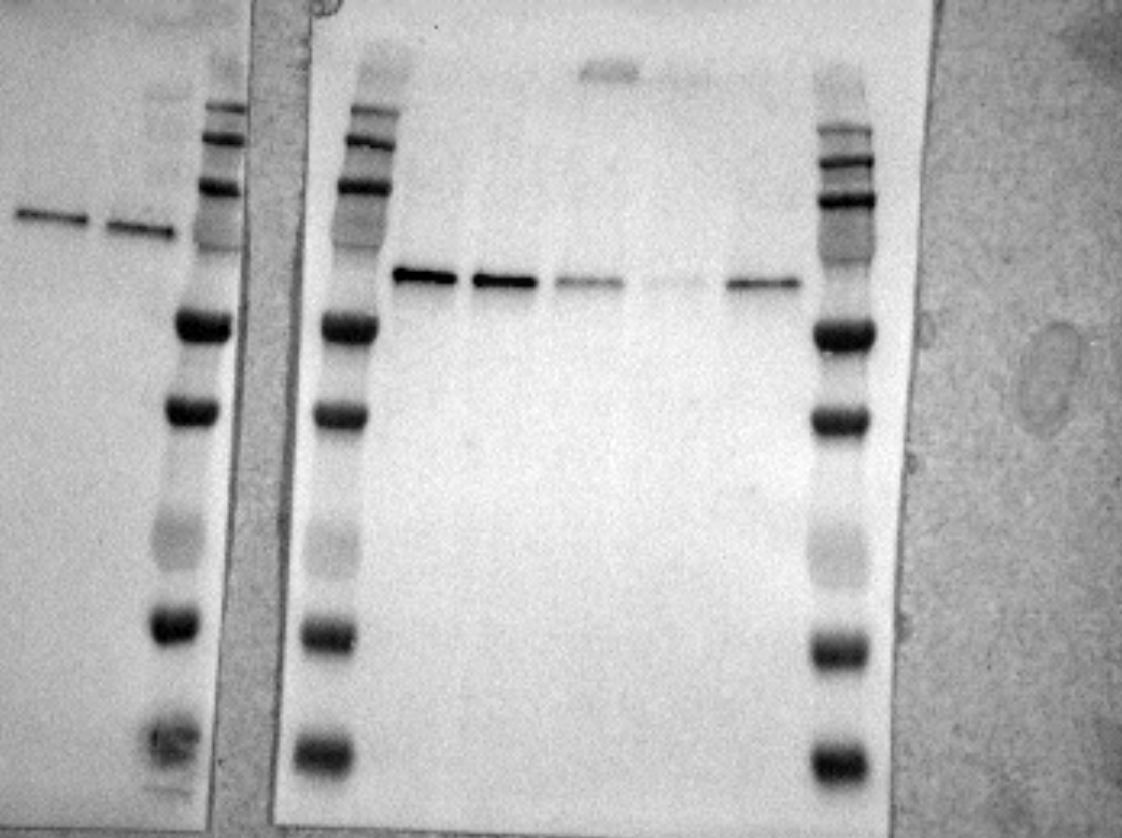

Supplement: Figure 1—figure supplement 1—source data 1. [file elife-89225-fig1-figsupp1-data1.zip › Figure 1-figure supplement 1D-source data 1 - Original files for western blot analysis displayed in Figure 1-figure supplement 1D/Figure 1-figure supplement 1D - Anti-RFP for HXK-3-mKate2.tif]

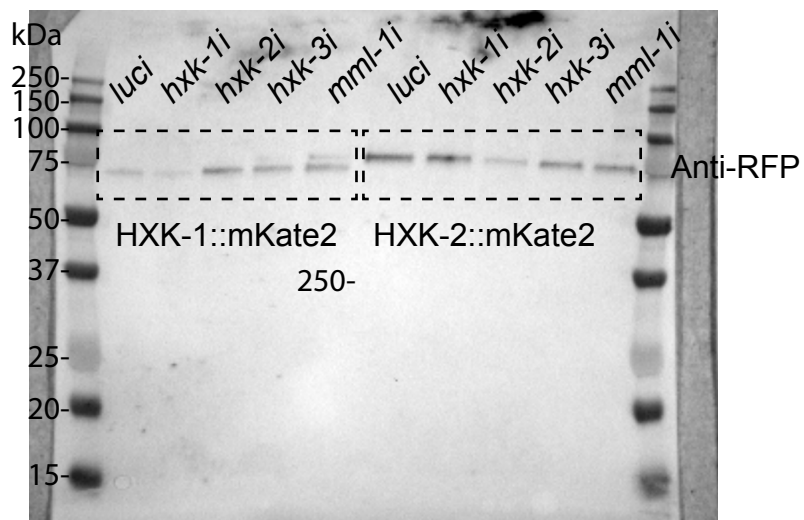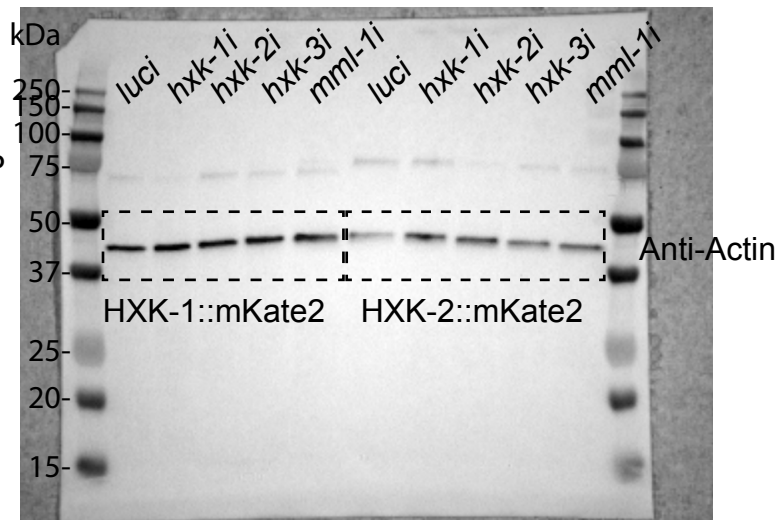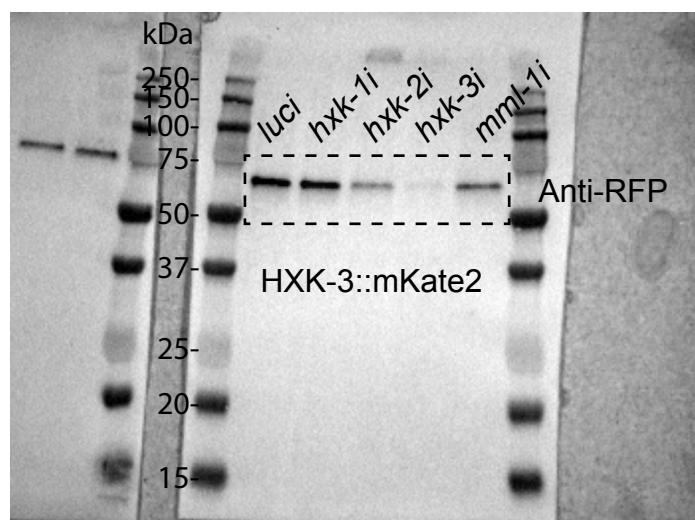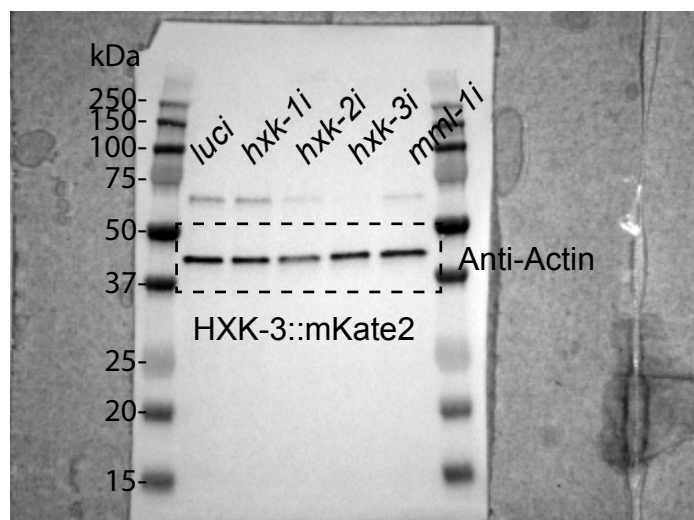

Supplement: Figure 1—figure supplement 1—source data 2. [file elife-89225-fig1-figsupp1-data2.zip › Figure 1-figure supplement 1D-source data 2 - Uncropped western blots with bands labeled displayed in Figure 1-figure supplement 1D/Figure 1-figure supplement 1D-source data 2.pdf]
